# Supplementary figures and images for: Neighbor-enhanced diffusivity in dense, cohesive cell populations
Source: PLoS Comput Biol. 2021 Sep 23;17(9):e1009447. doi: 10.1371/journal.pcbi.1009447 (PMC8491951; doi:10.1371/journal.pcbi.1009447)

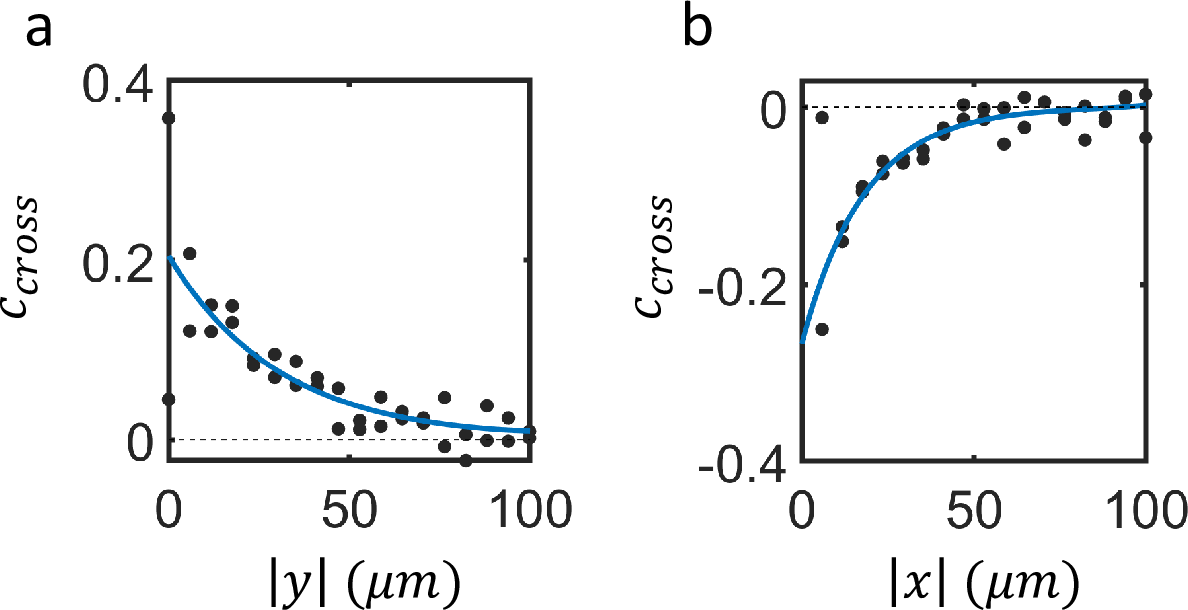

Supplement: S1 Fig — (a) Mean correlation function along the migration (y-) axis (temporal and ensemble mean). Exponential fit is drawn as blue solid line (correlation length, 30.75μm). (b) Correlation function along the axis perpendicular to the migration (x-) axis. The exponential fit had correlation length of 18.08μm. (TIF) [file pcbi.1009447.s001.tif]

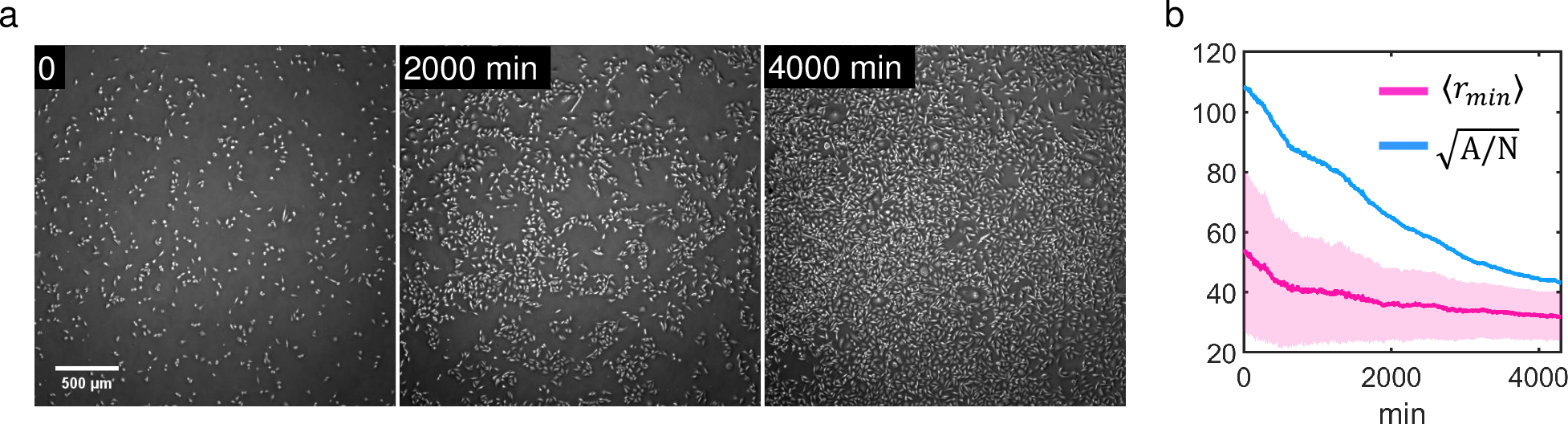

Supplement: S2 Fig — (a) Snapshot images acquired at different time. (b) Ensemble mean of minimum cell-to-cell distance 〈rmin〉 (red) and average inter-particle distance (blue), which is approximated as A/N where N is the number of cells, and A is the total area. The unit length is μm. This result illustrates that cells tend to adhere to each other forming small colonies; and this tendency becomes less pronounced as the cell population gets confluent. (TIF) [file pcbi.1009447.s002.tif]

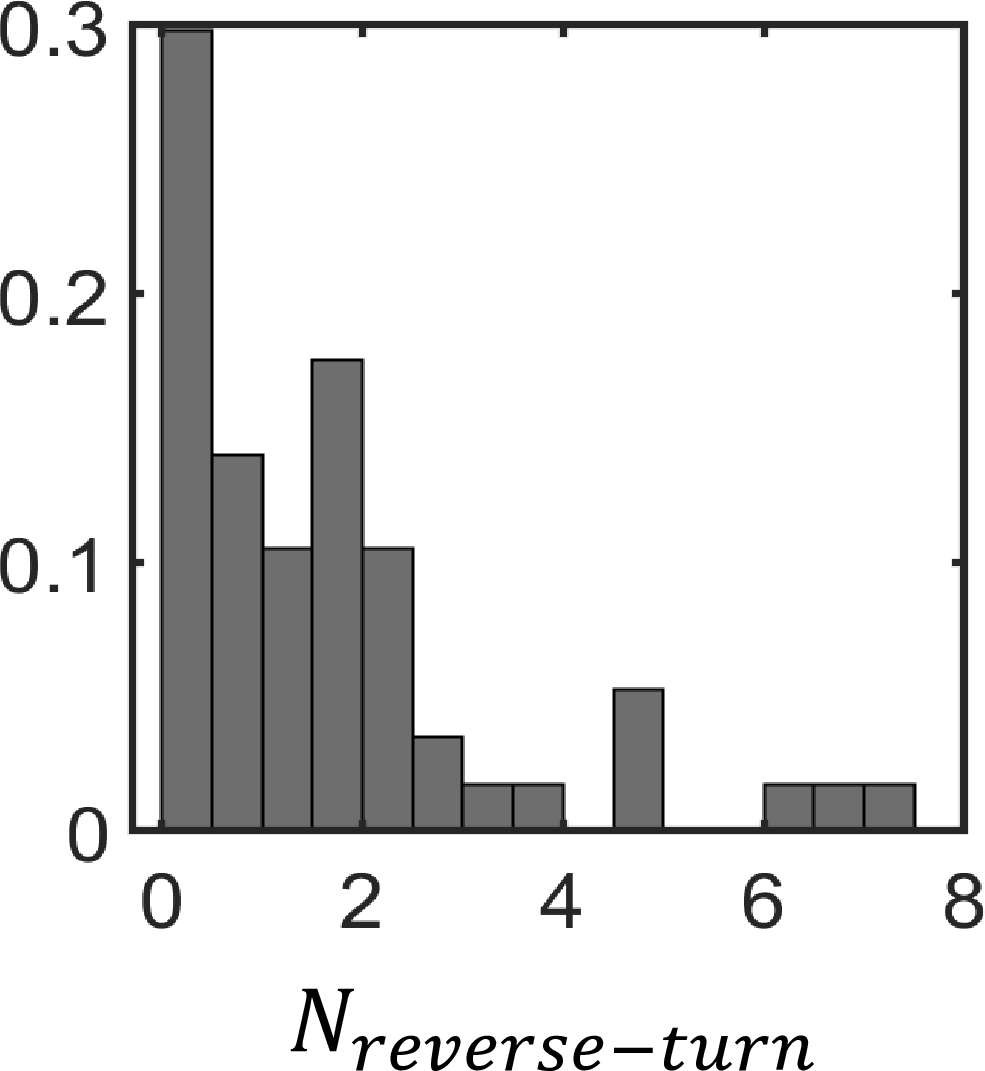

Supplement: S3 Fig — (TIF) [file pcbi.1009447.s003.tif]

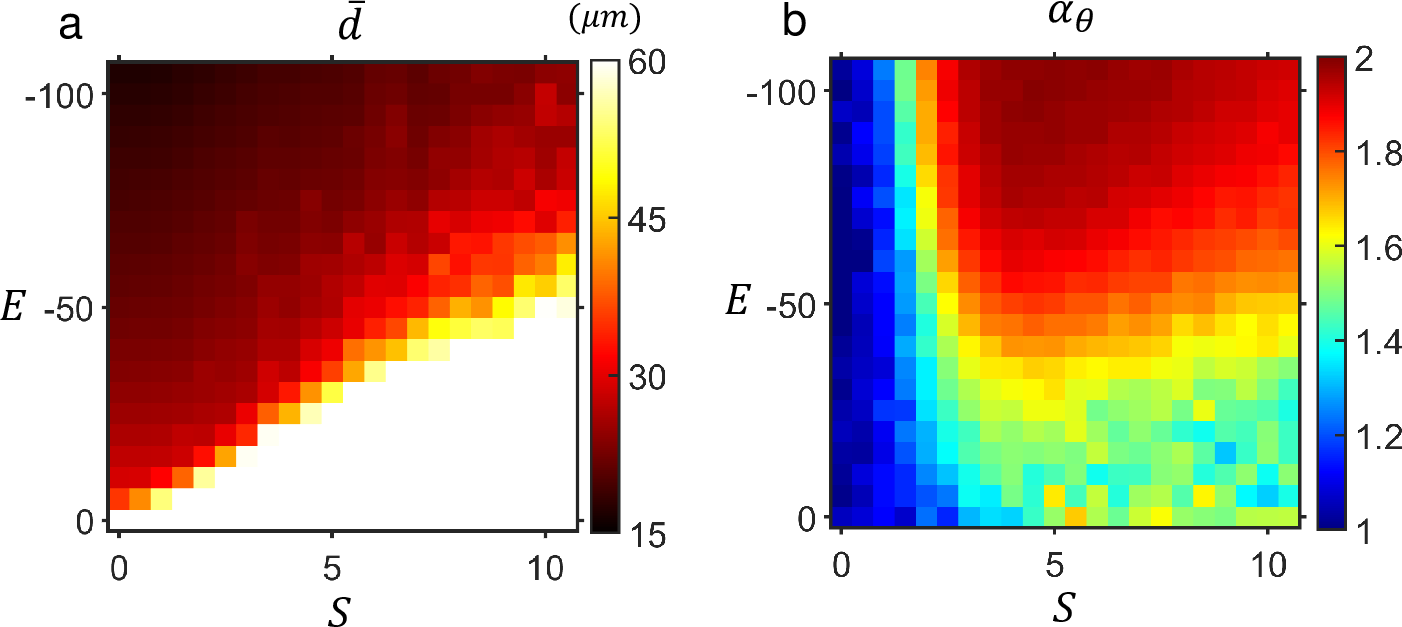

Supplement: S4 Fig — (a) d¯: Average distance between pair’s centroid and cells. d¯ was averaged temporally and over the ensemble of doublets. The white region represents where pairs separate due to strong propulsion overpowering the adhesion strength. (b) Diffusion exponents of angular diffusion (based on time domain: 70 ~ 100 min). For (a) and (b), 200 doublets were analyzed. (TIF) [file pcbi.1009447.s004.tif]

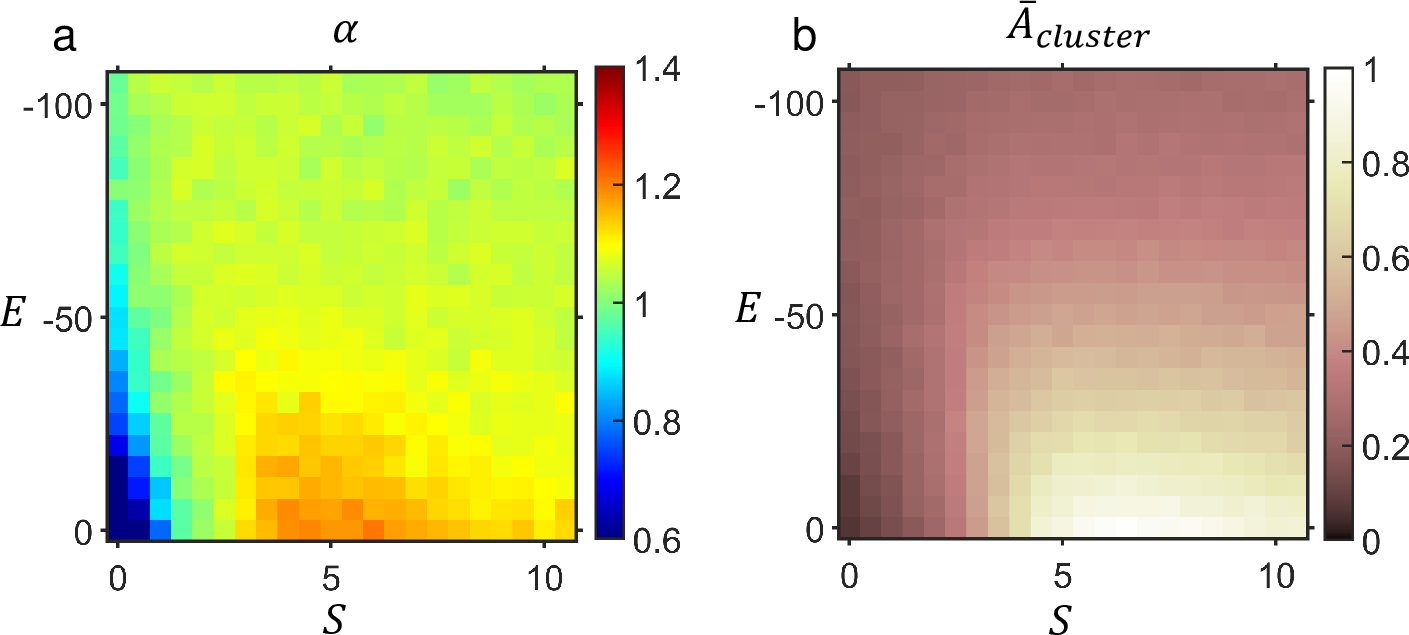

Supplement: S5 Fig — (a) Diffusion exponent α (based on the ensemble mean of MSD, in time domain of 400 ~ 600 min). (b) Normalized (temporal and ensemble) average cluster size A¯cluster. Cells belonging to the same cluster were obtained by recursively finding neighbors that satisfy two criteria (first, neighbors should be within 51 μm from a reference cell, and second, their velocity vectors should align with that of the reference cell within 20°). (TIF) [file pcbi.1009447.s005.tif]

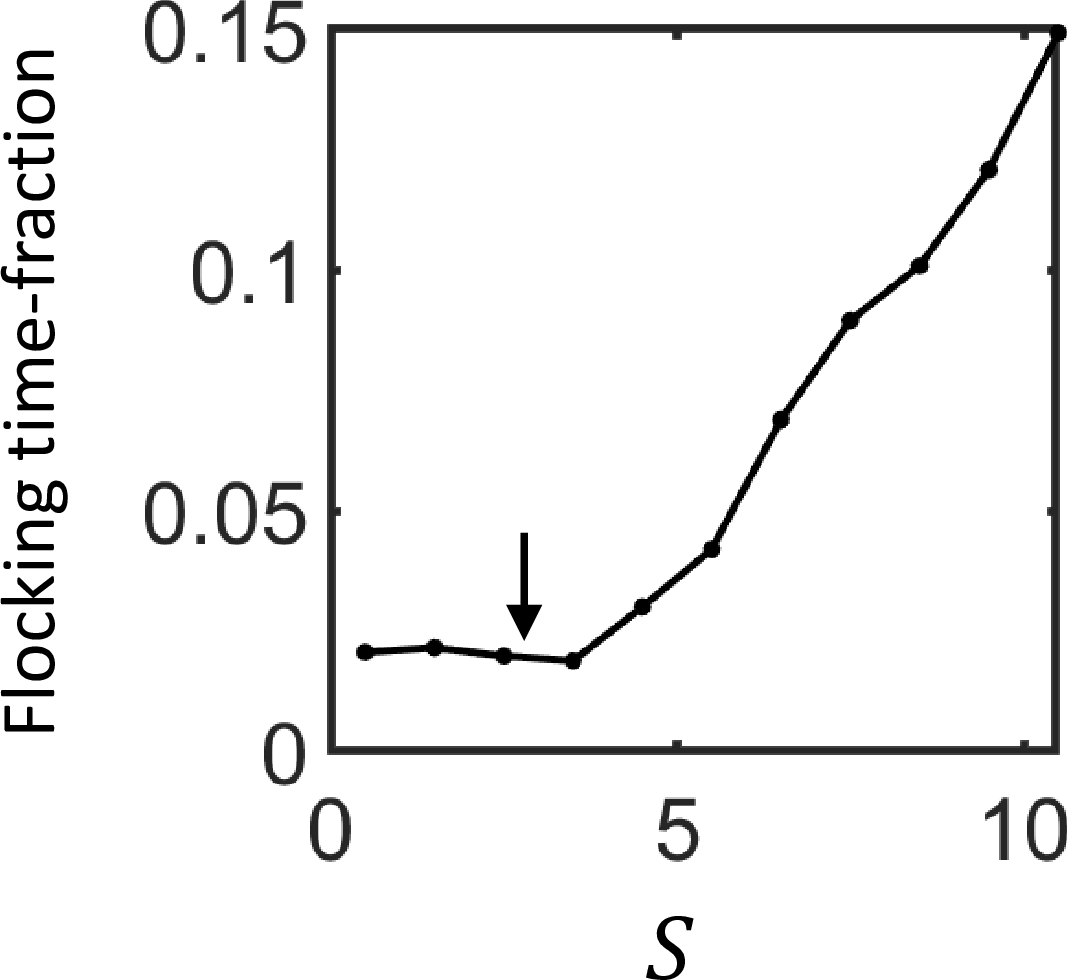

Supplement: S6 Fig — Criterion for the flocking mode was set as: 1) mean centroid speed > 0.45 μm/min and 2) mean angular speed < 0.01μm/rad. The arrow marks S = 2.8. (TIF) [file pcbi.1009447.s006.tif]

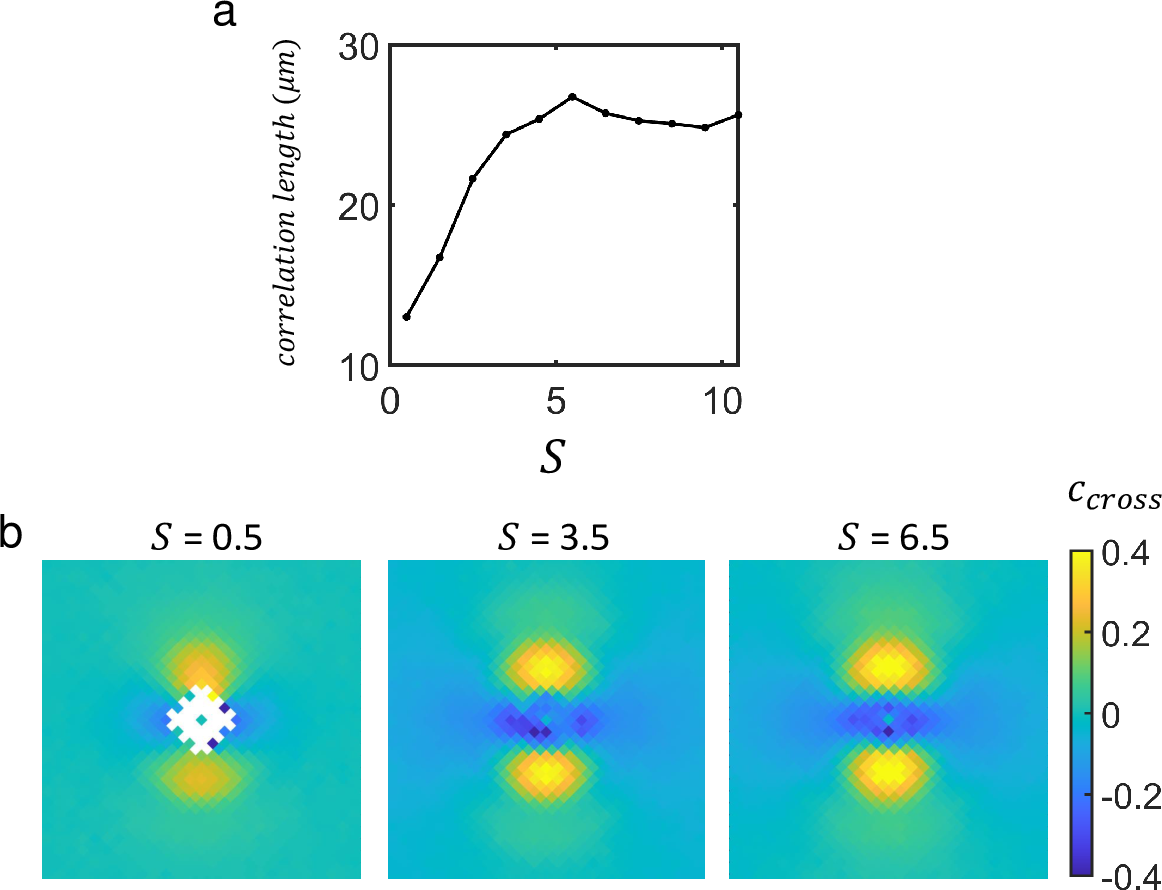

Supplement: S7 Fig — (a) Correlation length along the migration axis as a function of S, which was obtained by fitting the velocity-velocity correlation as in S1B Fig. (b) (Temporal and ensemble) mean velocity-velocity correlation maps for three different S. The averaging was done over 400 different reference cells and 60 different times. For each map, the width and height range from -80 μm to 80 μm with the reference cell at the center. The reference cell’s moving direction is aligned along positive y-axis. White-colored pixels represent where there have been no cell visits. (TIF) [file pcbi.1009447.s007.tif]

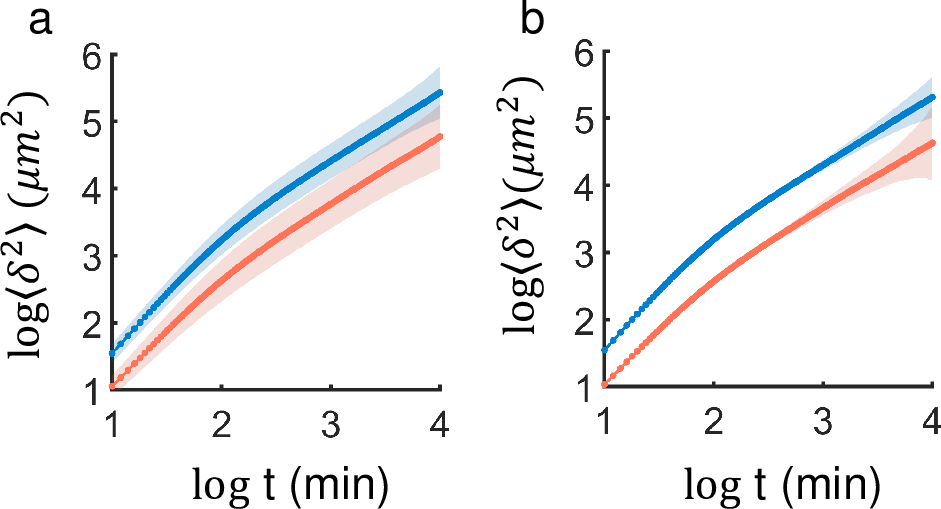

Supplement: S8 Fig — (a) S¯=2.8, σs = 1 with E = -65 fixed. (b) S = 2.8 fixed with 5 different types of cells + 1 medium, generating 15+1 different Es (uniformly distributed ranging from -55 to -75). Blue: confluent population; red: freely crawling cells. (TIF) [file pcbi.1009447.s008.tif]
