# Supplementary material for: Neighbor-enhanced diffusivity in dense, cohesive cell populations
Source: PLoS Comput Biol. 2021 Sep 23;17(9):e1009447. doi: 10.1371/journal.pcbi.1009447 (PMC8491951; doi:10.1371/journal.pcbi.1009447)
Supplement: Attachment — Submitted filename: ReviewComments.pdf [file pcbi.1009447.s017.pdf]

Review of PCOMPBIOL-D-21-00491 submitted to PLOS Computational Biology

The manuscript entitled "Neighbor-enhanced diffusivity in dense, cohesive cell populations" deals with how super-diffusivity of cells in a crowding situation is achieved. The authors first show that a cancer cell line exhibits a more diffusive profile in the confluent case than in the isolated case. Also, they report that not only multicellular swirling but also the continuous position swapping between neighboring cells in clusters of a few cells. Then, using the cellular Potts model, they show that a combination of cell-cell adhesion and active migration can generate explain their experimental observations. Their quantitative description of position swapping events in the cell clusters is somewhat interesting. However, analysis of mathematical model is less convincing. The main drawback of this manuscript is that there is no description of their mathematical model. Therefore, I am unable to judge whether this study meets the journal's criteria.

1) Please include details of the mathematical model. Otherwise, one cannot understand what the authors did, making impossible to judge the paper.

2) The main argument of this paper is whether the cell-cell adhesion enhances the super-diffusion of migrating cells. The authors used the cellular Potts model to show that the increase of the cell-cell adhesion parameter value leads to cell swirling in doublets and promotes the cell speed in the crowding condition in Figure 7. I am wondering whether these results are due to only cell-cell adhesiveness. Rather, I suspect that these changes stem from a stochastic aspect of the cellular Potts model, which results in flexibility of cell shape. What happens if you change values of parameters, such as temperature and a set of adhesion parameters? Can you reproduce the experimental observations using another model?

3) The authors claim that the diffusion exponent for the confluent cells was significantly larger than that of feely crawling cells shown in Figure 1c. However, the average values were almost the same between the two cases and their variances are very large. I recommend the authors perform statistical significance tests to support their claim.

Minor points:

On the data availability section, the authors state that all relevant data and codes are available

at github https://github.com/josephlee188/plos_comput_biol_data. However, there are no files in the repository. The authors should include the essential information for readers and reviewers.

P16, Line 414: “it has emerged in recent studies as an important structural signature for the migratory transition of a confluent tissue”. References are required.
