# Supplementary material for: Neighbor-enhanced diffusivity in dense, cohesive cell populations
Source: PLoS Comput Biol. 2021 Sep 23;17(9):e1009447. doi: 10.1371/journal.pcbi.1009447 (PMC8491951; doi:10.1371/journal.pcbi.1009447)
Supplement: Attachment — Submitted filename: Response_to_reviewers.docx [file pcbi.1009447.s018.docx]

Dear Editor,

We very much appreciate your effort in getting insightful and encouraging referee’s reports. Below, we answer and address all the questions and concerns of the referees in detail. Their comments and questions were valuable, and we believe that our manuscript is improved with the subsequent revisions.

Thank you

Sincerely,

Kyoung J. Lee

----------------------------------------------------------------------

List of major changes made:

1. 8 references [26, 32, 36, 37, 44, 45] are newly added.
2. 4 new supplementary figures are added (S1, S6, S7, S8 Figs).
3. Changes in the labels for supplementary figures: S1 🡪 S2; S2 🡪 S3; S3 🡪 S4; S4 🡪 S5 Figs.
4. In Figs 1c, 1d, and 4c, the labels for the y-axes (mean squared displacement) are fixed to “$\log\left\langle\delta^{2} \right\rangle(\mu m^{2})$” from “$\log\delta^{2} (\mu m^{2})$”.
5. Fig 1d’s time-range is now 10^2.2^ ~ 10^3^ min.
6. In Fig 3e’s lower right panel, $N_{normal}$ is fixed to $N_{rim}$.
7. In Fig 3f, the expression ‘Normal direction’ is fixed to ‘Direction along cluster rim’.
8. In Fig 6e’s lower right panel, $N_{normal}$ is fixed to $N_{rim}$.
9. Big parts of 2^nd^ and 3^rd^ paragraphs in the Introduction are deleted, and the paragraphs are combined into one with a better clarification (lines 62-76).
10. We add new lines regarding correlation lengths of the experimental cells within confluent population (lines 170-175).
11. We add a new paragraph concerning the transition between co-movement (flocking) and rotation in doublets with varying parameter. It also describes how velocity-velocity correlation changes in this parameter range (lines 436-448).
12. Discussion was significantly modified with new comments on 1) potential mechanism of the enhanced diffusivity in relation to doublet rotation (lines 458-465); 2) relevant earlier studies on rotational behavior of cell-clusters (lines 475-485); 3) relevant earlier reports on the enhanced diffusivity due to cell-cell contact interaction, and the distinction from our observation (lines 497-507); 4) contribution of the heterogeneity in cell population using distributed parameters in CPM simulations (lines 517-526).
13. Included a more detailed description of the computational model in Methods section (lines 552-575).
14. We provide captions for each newly added supplementary figure.

----------------------------------------------------------------------

**Reviewer #1**: The authors present an interesting study, including experimental and simulation data. However, I have several problems that they need to address:

1) There are some missing relevant refs:

Rotations of cell collectives in confinement:

Doxzen, Kevin, et al. "Guidance of collective cell migration by substrate geometry." Integrative biology 5.8 (2013): 1026-1035.‏

Segerer, Felix J., et al. "Emergence and persistence of collective cell migration on small circular micropatterns." Physical review letters 114.22 (2015): 228102.

For freely rotating cellular clusters:

Malet-Engra, Gema, et al. "Collective cell motility promotes chemotactic prowess and resistance to chemorepulsion." Current Biology 25.2 (2015): 242-250.‏

Copenhagen, Katherine, et al. "Frustration-induced phases in migrating cell clusters." Science advances 4.9 (2018): eaar8483.‏‏

**Reply #1-1:**	Thank you. In the revision, the four suggested references are cited and discussed (lines 475-485).

2) It’s not clear if the characterization of the dynamics by the MSD exponent alpha is very meaningful in the sense of diffusion-vs-super-diffusion, since even a persistent RW eventually gives rise to normal diffusion at long enough time scales.

**Reply #1-2:**	The (super-diffusion-to-diffusion) transition was discussed already with Fig 1d (and lines 117~121 in the revision). What is significant and interesting comes before this transition. Our analysis shows that 1) the diffusion exponent $\alpha$ is larger for the cells in confluency (as versus to freely crawling cells) and 2) the transition comes at a significantly later time (501 min) for the cells in confluency in comparison to 251 min of freely crawling cells.

3) On page 7 they discuss the cell migration. In addition to the analysis that they present, they should plot the velocity-velocity correlations and extract the correlation length, as done for example in [4].

**Reply #1-3:**	A new supplementary figure (S1 Fig) shows velocity-velocity correlation as suggested. MDA-MB-231 cells in this study exhibit azimuthally non-uniform velocity-velocity correlation (see Fig1f and Fig4f). Therefore, we have measured spatial correlation length along the vertical axis (i.e., the line of cell polarization) and that along the horizontal axis separately.

We added a comment on this result in the revision (lines 170-175).

4) They write that: “neighbors have a tendency of moving in the opposite direction of the reference cell is heading.”, so do they move in “lanes” ?

They could try the analysis in terms of “co-moving cell clusters”, performed in: Zaritsky, Assaf, et al. “Propagating waves of directionality and coordination orchestrate collective cell migration.” PloS Comput Biol 10.7 (2014): e1003747.‏

**Reply #1-4:**	First of all, we point out that the correlation map for the experiment (Fig 1f) and that of the simulation (Fig 4f) are a time- and population-averaged picture. The maps illuminate, on average, that local interaction generates 2 ~ 3 co-moving cells along the y-axis (i.e., instantaneous axis of moving direction of a reference cell placed at the origin) and ~1 cell moving in the opposite direction at both sides of the reference cell. In addition, the average size of co-moving cluster was also analyzed and given in S5b Fig.

In a significant range of the 2D ($E$ and $S$) parameter space, cell-doublets can exhibit two different motility modes inter-changeably in time: co-moving (flocking) and rotating behaviors. In a co-moving or flocking mode, they move in line like a two-cart vehicle. Depending on the relative strength of cell-cell adhesion and that of self-propulsion, the time-fraction in which co-moving mode occurs varies. For example, for a fixed cell-cell adhesion energy $E$ = -65, increasing the self-propulsion strength $S$, a continuous transition to the flocking from the rotation mode takes place (see our new S6 Fig). Interestingly, the experimentally plausible value $S$ = 2.8 (see Fig 7e) lies below the critical value (of $S$ ~ 3.5) for the transition; subsequently, for cell-doublets rotation mode would dominate. Meanwhile, correlation length along the migration axis (S7a Fig) also displayed a transition around the same parameter value, while in this case the correlation length saturated to around a single-cell’s diameter. On the other hand, oppositely moving neighbors along the horizontal axis (both sides of the reference cell in S7b Fig) implied that there is remnant of the rotating pairs even within confluent population.

We provided a new paragraph on this issue in lines 436-448.

5) Could it be that the observed increase in motility when cells interact is related to large flows induced by confluence in cellular nematics? especially as their cells are highly elongated.

See:

Duclos, G., et al. “Spontaneous shear flow in confined cellular nematics.” Nature physics 14.7 (2018): 728-732.‏

Duclos, Guillaume, et al. “Perfect nematic order in confined monolayers of spindle-shaped cells.” Soft matter 10.14 (2014): 2346-2353.

**Reply #1-5:** As noted in our Reply #1-3, the correlation lengths along the vertical (along the moving direction) as well as horizontal (perpendicular to the moving direction) axis were of scales of a few or about a single cell-dimeter (30.75 and 18.08 $\mu m$, respectively). Therefore, there is no large-scale cellular flows in our preparations. However, as we commented in lines 461 and 462, we postulated that the enhancement of diffusivity is due to the (albeit short-ranged) correlated movement of neighbor along the target cell’s moving direction.

6) On page 8 a “modulation period tau_d” is introduced but not defined or explained. Is it the mean time between changes in the direction of rotation? Ie rotational persistence time ? should be clearly explained.

**Reply #1-6:** Thank you. We have clarified the definition of $\tau_{d}$ in the revision (lines 211-212).

7) Are the doublets ever breaking up ? if they do, what is their life-time distribution ? ‏

**Reply #1-7:** The observed doublets rarely separated unless the constituent cells accidentally came in contact with other cells, at which they were no longer considered as a doublet and excluded from our analysis.

8) Points 6,7 also refer to the quadruplets.

**Reply #1-8:** We clarified the definition of $\tau_{d}$ for quadruplets in lines 263 and 264.

Same was true for the quadruplets as in comment 7: isolated quadruplets almost never separated. We extracted only the parts in which they formed adhesion with themselves, and no cell-division occurred.

9) On page 11 the cell swapping is described along the "normal direction", but a better term is "nearest neighbors along the cluster rim".

**Reply #1-9:** Thank you. The term was revised (lines 264-265).

10) The biggest problem in this work is the simulations:

It is not explained why in these simulations do they find the observed behavior.

What is the mechanism in the simulations that gives rise to larger diffusivity in confluency? and larger persistence length and time for the single cell trajectory? using the simulation they should be able to pinpoint the mechanism, but I don’t see it explained anywhere.

Similarly, they do not explain why the rotations arise in their simulations.

**Reply #1-10:**

Our investigation has begun from the experimental observation (and analysis) that the cells in confluency have a larger persistence length (and time) and better super-diffusivity. Incidentally, we also observed the quite robust doublet rotation phenomenon. In order to elucidate these two interesting (seeming unrelated) phenomena, we have carried out a systematic investigation and data analysis both for experiments and model simulation. For recapitulating all of our experimental observations, we find two features are essential and they seem to be sufficient: namely, 1) self-propulsion (tuned by $S$) and 2) cell-cell adhesion (tuned by $E$). We could tune the set of ($S$, $E$) to realize various modes of cell motility as shown in Fig 7. What is significant of our work is that we could narrow down to a particular set of ($S$, $E$) with which we recapitulated very much everything (not only for the cells in confluency but also for the doublets and quadruplets) that we observed in experiments. Pinning down to a particular set of ($S$, $E$) was possible, as we measured various properties like $\left\langle corr_{pair} \right\rangle$, $\left\langle r \right\rangle$, $\bar{v}$, and $\bar{p}$ in experiments and asked for our simulations that would match them simultaneously. That is the main purpose of Fig 7. We believe this multitude requirement check is noble and not just a simple parameter fitting.

After all, the observed phenomena can be realized if we have cells that have a proper degree of directional persistence and are just sticky enough to hold on to each other. For the selected set of ($S$, $E$), doublets rotate about each other. But in other regime the doublets can be co-moving or flocking (a circular rotation is unstable in this regime). The transition between the two different modes is continuous as discussed in Reply #1-4. The enhanced directional persistence and super-diffusivity in confluency is a consequence of acquiring a small but finite velocity-velocity correlation length along the direction of instantaneous moving direction. As already discussed in Reply #1-4, the small correlation length along the y-axis is a reminiscence of the co-moving doublet forming a “two-cart vehicle.” So, on average, the cell motility in confluency can be viewed as a random mixture of cell doublets co-moving or rotating together in a time-shared manner.

11) The discussion of Fig.7 is very descriptive. No mechanism is explained, and the region which fits the experiments should be indicated on the figures.

The simulations do not provide us with any understanding of the mechanisms that give rise to the experimental observations, and how these depend on the physical properties of the model, and why.

This is the main purpose of using theoretical simulations, and is lacking here.

**Reply #1-11:** Please see our Reply #1-4 and #1-10 alongside our additional supplementary figures and discussions.

**Reviewer #2**: The review comment is uploaded as an attachment.

The manuscript entitled “Neighbor-enhanced diffusivity in dense, cohesive cell populations” deals with how super-diffusivity of cells in a crowding situation is achieved. The authors first show that a cancer cell line exhibits a more diffusive profile in the confluent case than in the isolated case. Also, they report that not only multicellular swirling but also the continuous position swapping between neighboring cells in clusters of a few cells. Then, using the cellular Potts model, they show that a combination of cell-cell adhesion and active migration can generate explain their experimental observations. Their quantitative description of position swapping events in the cell clusters is somewhat interesting. However, analysis of mathematical model is less convincing. The main drawback of this manuscript is that there is no description of their mathematical model. Therefore, I am unable to judge whether this study meets the journal’s criteria.

1) Please include details of the mathematical model. Otherwise, one cannot understand what the authors did, making impossible to judge the paper.

**Reply #2-1:** We added more detailed description of the model in lines 552-575.

2) The main argument of this paper is whether the cell-cell adhesion enhances the super- diffusion of migrating cells. The authors used the cellular Potts model to show that the increase of the cell-cell adhesion parameter value leads to cell swirling in doublets and promotes the cell speed in the crowding condition in Figure 7. I am wondering whether these results are due to only cell-cell adhesiveness. Rather, I suspect that these changes stem from a stochastic aspect of the cellular Potts model, which results in flexibility of cell shape. What happens if you change values of parameters, such as temperature and a set of adhesion parameters?

**Reply #2-2-1:** Thank you. Of course, the effective temperature T in CPM is an important parameter. We would expect that the higher the value of T is, the more severe random fluctuations for all different interfaces defining cell boundaries would experience, which would relatively diminish the significance of any directed motion of the cells. To confirm this, we ran CPM simulations with widely different temperature values (otherwise, keeping the same set of parameter values used for Figs 4-6). Increasing the temperature from 0.1 to 100 (T = 10 was used throughout the paper), the model cells (both in confluent and freely crawling) gradually became normally diffusive. Interestingly, single-cells approached normal diffusion faster than the cells in confluency did. In the case of cell-doublets, robustly rotating pair stopped showing rotational movement beyond a critical temperature around 20. So, we confirmed that high stochasticity disrupts the persistent movement of cells.

Can you reproduce the experimental observations using another model?

**Reply #2-2-2:** We believe the same phenomena can also be reproduced by other agent-based models as long as they implement cell-shape deformation, a proper cell-cell adhesive interaction, and self-propelling ability. The main reason we have used CPM is its simplicity.

3) The authors claim that the diffusion exponent for the confluent cells was significantly larger than that of feely crawling cells shown in Figure 1c. However, the average values were almost the same between the two cases and their variances are very large. I recommend the authors perform statistical significance tests to support their claim.

**Reply #2-3:** Although the cell-cell variances are quite large (an intrinsic property of MDA-MB-231), which is a mesoscopic phenomenon understood at the single-gene level. For the time domain of interest (log158=2.2 ~ log500 = 2.7 in Fig. 1d), the average $\bar{\alpha}_{free}=$ 1.07 < $\bar{\alpha}_{confluent}=$ 1.24 and the difference is statistically very significant (one-way ANOVA test p < 0.001). In fact, given the maximum value of $\alpha=2$(for a ballistic motion), the difference $\Delta\alpha= \bar{\alpha}_{confluent}- \bar{\alpha}_{free}=$ 0.17 is quite meaningful.

We added the corresponding statements in Fig 1’s caption (lines 132-134).

**Minor points:**

On the data availability section, the authors state that all relevant data and codes are available at github https://github.com/josephlee188/plos_comput_biol_data. However, there are no files in the repository. The authors should include the essential information for readers and reviewers.

🡪 We plan to upload a final set of data and code files once the editorial process is completed.

P16, Line 414: “it has emerged in recent studies as an important structural signature for the migratory transition of a confluent tissue”. References are required.

🡪 Thank you. We added two references in the revision (ref. no 5 and 7) in line 427.

**Reviewer #3**: The authors present a cell-tracking study of MDA-MB-231 cells densely packed in 2-dimensional layer.

The cell trajectories are studied with well-established statistical methods in the field, such as MSD, and time-spatial correlations. They report a neighbor-enhanced diffusivity phenomenon where paired neighbor-cells exhibit a higher diffusivity than those of freely moving single cells. Further, they observe cell-doublet and cell-quadruplet rotation. Interesting, the authors were able to reproduce these dynamic properties with a Potts Model by tuning only two parameters: adhesiveness and self-propulsion. I found the findings very interesting. the fact that two parameters of the CPM can account the phenomenon is very instructive about the processes underlying these dynamic properties. However, there are several issues that need to be addressed prior to its recommendation. I will list my concerns below

**🡪** We thank the referee for his/her encouraging comments and lucid summary on our work.

Major issues

1.- The present form of the Introduction is no appropriate. For example: the text about biological transitions (2nd and 3rd paragraphs lines 64-88) is not well connected with the present results.

It is enough to mention: " ... biological tissues are a collection of interacting ‘active’ cells in non-equilibrium states, therefore, in principle, cell-population can support numerous different states, to which an initially sedentary tissue state can switch." and appropriate refs.

I suggest that the authors include a discussion that sheds light on the title, for example, what are the differences between NED and CEL that justify a new category?

**Reply #3-1:** First of all, we combined the previous 2^nd^ & 3^rd^ paragraphs of Introduction and kept the only the essential part as suggested in the revision.

We extended our discussion on CEL (as versus to NED) in the revision (lines 497-507).

2.- The term super-diffusivity must be restricted to the observational time-scale. If the authors calculate MSD with a larger number of MC steps in the simulation, they can see that the Brownian motion is replaced by the ballistic regime It is documented in doi:10.1103/PhysRevE.101.062408 and 10.1103/PhysRevLett.99.010602

The authors must be more rigorous with these terms all over the text.

**Reply #3-2:** Thank you

We have used the term “super-diffusivity” somewhat loosely. According to [32], one may require that $\alpha>1$for the time greater than the directional persistence $\tau_{2}$ as a necessary condition for super-diffusivity. We measured the transition time point to normal diffusion to be 251 min (for freely crawling cells) and 501 min (for cells in confluency), which are much larger than $\langle\tau_{2,free}\rangle=$66 min and $\langle\tau_{2, pop}\rangle=$ 116 min, respectively. Thus, it seems appropriate to use the term “super-diffusivity” in our case.

We have added these statements in the revision (lines 154-159).

3.- In lines 429-432, Even in a monoclonal cell population it is expected and heterogeneous. This variability is due to intrinsic and extrinsic stochastic gene expression, a mesoscopic phenomenon well understood at the single-gene level. The sentence in line 432 is not very precise. Moreover, despite heterogeneous behavior on cell culture, the authors use a "homogeneous" in-silico cells. I suggest to authors to use a distributed parameter values in the simulation. i.e., each cell in the CPM have intrinsic value of S, and the same in other simulation regarding the E parameter. In this manner, one can understood the contribution of adhesiveness and/or self-propulsion to the observed variability.

**Reply 3-3:** First of all, we thank referee #3 for letting us know of the potential biological origins for the observed variability. We have clarified the part (lines 517-520).

Also, we carried out additional simulations 1) with heterogeneous distribution of $S$ for a fixed value of $E$ (as shown in the new supplementary figure S8a Fig) and 2) with 5 different types of cells + 1 medium, generating 15+1 different $E$s for a fixed value of $S$ (S8b Fig), to see which parameter has a more significant contribution to the observed variability. We find that the variability in the self-propulsion (i.e., $S$) is more significant, and we discussed this finding in the Discussion section (lines 517-526).

4.- In lines 442-446, the authors discuss previous finding regarding the role of cell-to-cell adhesion in the phenomenon. There is a previous paper which is in line with the present finding (NED) and the role of cell-to-cell adhesion (Fig.2 and 7 of Physica A 365 (2006) 481-490). The paper analysis the cell locomotion of cell that express MOCA protein a protein that change cell adhesion by regulating the activity of Rac1 and N-cadherin. The author must to add to the Discussion and Introduction (see point 1)

**Reply 3-4:**	Thank you for the information.

We cited the new reference [26] at the end of our revised Introduction and discussed it in Discussion (lines 497-501).

The velocity-velocity spatial correlation function (in Fig.4a of [26]) shows a correlation length about the size of a single cell. Interestingly, it appears that this correlation function was obtained while the cells were having random contacts but not in a confluency. So, the situation seems to be more akin to the case of CEL which does not require cell-cell adhesion.

The velocity-velocity correlation profiles for the MDA-MB-231 and the simulated confluent cells (Fig 1f and Fig 4f) displayed non-uniform azimuthal distribution with respect to the reference cell’s moving direction, and the correlation lengths for both vertical (positive correlation) and horizontal (negative correlation) only amounted to about a single cell’s diameter (as in [26]).

Minors

a.- The paper emphasises on the fact NED s counter-intuitive dynamic property, I am not agree.

🡪 We restrained using the word “counter-intuitive” throughout the paper.

b.- The sentence: "The study of Mitchel et al. is an excellent example suggesting that the relevant phase space for the dense cell population needs to be at least two-dimensional. " What two dimensions are you referring to?

🡪 It means that to “cover” all the observed phenomena (i.e., dynamic states), it requires at least two independent parameters. We revised the relevant sentence for a better clarity (line 76).

c.- The sentence: "With phenomena like ‘contact inhibition of locomotion’ and ‘volume exclusion’ "

is wrong, "volume exclusion" is not a phenomenon.

🡪 We removed the use of “volume exclusion”.

d.- I don't understand the lines 46-47.

🡪 We edited the lines for clarification.

e.- I feel that the intermittent position change events in quadruplets are collateral,

they do not represent an intrinsic phenomenon, and something is oversized in the text.

🡪 In general, we can expect a few different modes for a quadruplet including a rigid body-like rotation. For the case of MDA-MB-231 cell quadruplets, a rigid body-like rotation seems to be a preferred dynamic feature but there comes some occasional pairwise position swapping events, which are a reminiscence of cell doublet rotation. As the referee suggests the pairwise position swapping events may be viewed as collateral, but what is important is that the particular set of ($S$, $E$) used for CPM simulations throughout the whole paper would also produce such events. So, at least this collateral phenomenon gives a strong support of the CPM and the particular set of ($S$, $E$) that we have employed for the elucidation of our experimental findings.

Why the authors use the self-propulsion proposed by Szabo et al. instead of the one proposed in 10.3389/fphy.2018.00061?

🡪 Thank you for letting us know of the paper by Guisoni, et al. (2018), which discuss a CPM model that is very similar to (but slightly different from) the one that we have used. As far as we understand, only a major difference lies on how to implement the directional persistence (i.e. memory) into the moving cell. In our case, there is only one time constant related the directional memory, but in the case of Guisoni’s model there seems to be an additional time constant, which is related to the probability of changing the direction of the force at each Monte Carlo update. We have not done any simulations with the model of Guisoni et al. but expect to obtain similar results.
