# Supplementary material for: Neighbor-enhanced diffusivity in dense, cohesive cell populations
Source: PLoS Comput Biol. 2021 Sep 23;17(9):e1009447. doi: 10.1371/journal.pcbi.1009447 (PMC8491951; doi:10.1371/journal.pcbi.1009447)
Supplement: Attachment — Submitted filename: Response_to_reviewers.docx [file pcbi.1009447.s019.docx]

List of major changes made:

1. Figure caption of Fig 7 is rewritten with further details for a better clarity (lines 413-430).
2. Fig 8 and its figure caption are newly added (lines 526-538).
3. In connection to Fig 8, we added a new paragraph on how (in the model) interfacial deformation could be amplified, leading to rotational motion (lines 515-524).
4. Two new references [48,49] are added with discussion.
5. Added a new paragraph on the similarity between graded motility in [37] and the quadruplet motility in Discussion (lines 496-501).
6. Added new comments on the limitation of our computational model in Discussion (lines 510-514, 540-558).

----------------------------------------------------------------------

**Replies to Comments to the Authors (2^nd^ round):**

Reviewer #1: The authors have addressed the comments, but some are still in need for clarification, as I list below.

1) The caption for Fig.7 should be expanded, with each panel explained, what are the lines, what is the colorbar indicating etc.

Answer #1-1: We have expanded the figure caption of Fig 7 for a better clarity (lines 413-430).

2) The computational model does not include any effect of contact-inhibition of locomotion (CIL) or any tendency for cells to align their polarization with neighbors. Both effects were demonstrated in many multi-cellular systems, so they need to explain and emphasize this fact, and why in these cells they think these interactions are not present.

Answer #1-2:

The referee is right that our CPM does not have a built-in CIL mechanism, where CIL refers to the phenomenon in which the physical contact between (colliding) two (more more) cells initiates some chemical reactions that affect either the mechanics or the adhesive property of the cells involved. As discussed in the two earlier (phase field) modeling papers [46, 47], CIL could be a critical element, as important as cell mechanics (eg. shapes), propulsion strength, cell polarity, and adhesion, as for governing the behavior of cells in contact, an example being the rotation of a cell doublet. Our CPM has all other elements but CIL. Unfortunately, we do not know if MDA-MB-231 cells have any significant CIL and if it they do how they work. (We would be happy to have them and incorporate them into our CPM.) The same is true for cell polarization: Simply, we do not know if there exists any “direct” polarization-polarization interaction among neighbors in our MDA-MB-231 populations.

In our model, however, there is a built-in polarization vector – velocity alignment tendency and the polarization (velocity) vectors interact indirectly by the cell population dynamics (i.e., cellular contacts): Each polarization vector changes as the cell’s centroid position, which is affected by the shape of the cell that are influenced by its adjacent neighbors, moves in space.

Again, what seems to be the most significant of our CPM study is that without having any complex CIL or direct P-P interactions, the toy model can still well reproduce all n=1, n=2, n=4, and n→∞ phenomena rather well in a consistent way, in particular, based on a single set of parameters ($S$, $E$). For the future, it will be worthwhile to add on additional elements (such as CIL or direct P-P interactions) in the CPM or other types of models in which the polarity vector P is not given but naturally determined by the internal dynamics.

We added comments regarding these issues in the revision (lines 510-524).

3) The model shows that where two adhered cells maintain contact, but this contact does not affect their polarization, they rotate. This is quite trivial, considering that two self-propelled particles, even described as point-particles (i.e., without the CPM detailed description of the cell shapes), cannot do anything else under such conditions. The strong binding into a pair, which has to rotate unless the forces are aligned, has therefore a similar effect to the external confinement shown in refs.44,45. They should discuss these issues at greater depth.

Answer #1-3:

We believe there was a misunderstanding. Please see our Answer #1-2: The polarization vectors of two neighboring cells in our CPM interact one another indirectly via their contact interface.

With the indirect interaction along with other factors, various [gliding (running), wobbling (random), or rotating] modes are possible as depicted in the diagram of Fig. 7. It turns out that for the case of MDA-MB-231 and the relevant CPM simulation, the robust rotation is a preferred state over gliding or wobbling modes and this happens with a good degree of cell-cell adhesion. In that sense, our cell doublets having open boundaries could be similar with the cell doublets within a small confinement as discussed in refs 44 and 45. Of course, no gliding or mixed mode will be possible within small confinements.

Now, we discuss the emergence of doublet rotation in a greater detail with an additional figure (Fig. 8).

4) The CPM model that is used contains a self-propelled polarization vector that is not emergent from the CPM-calculated cell shape, but is external to it. There is not dependence of the polarization on the cell shape. This makes this simulation rather simple-minded. See for example a variant of the CPM where polarization and persistence emerge from the underling dynamics, and therefore motility and persistence are highly shape dependent, which should be mentioned in the discussion of the model that is used and its limitations:

Niculescu, Ioana, Johannes Textor, and Rob J. De Boer. "Crawling and gliding: a computational model for shape-driven cell migration." PLoS Comput Biol 11.10 (2015): e1004280.‏

Wortel, Inge MN, et al. "Local actin dynamics couple speed and persistence in a Cellular Potts Model of cell migration." Biophysical Journal (2021).‏

Answer #1-4:

Again, the polarization vector for our model cell is not emergent directly from the cell shape; yet, its evolution depends on the cell centroid position, which evolves according to the cell shape. For the moment, it is not clear how critical to have the polarization vector evaluated from the cell shape directly. So, for the future project, it would be interesting to simulate the more realistic CPMs that are based on realistic actin dynamics inside the cytosol and polarization interactions. We thank the referee for raising this issue.

We discussed about the limitations of our model and the suggested references in the revision (lines 540-558).

5) If I understand the model, along the pixels where cells are adhered to each other, both cells are less likely to make self-propelled moves forward. So these regions are essentially less motile, compared to the outer edges along the outer side of the doublet. If this is true, you have a graded motility that resembles the rim-bulk graded motility that was shown in [37] to give rise to rotating clusters. This can be explained.

In general, I think the authors added references to previous works, but did not attempt to closely compare their model to those previous works. I think they should make an effort to make these comparisons, it will benefit the readers.

Answer #1-5:

As we understand, the picture of “graded motility” was employed at the spatial resolution of cells forming a cluster [37]; thus, it is unclear how the picture of pre-assigned, space-dependent, strength of cell motility persistence can be translated for the pixels comprising CPM cells.

For the mechanism underlying the doublet rotation, please see our Answer #1-3: Again, we have added a new figure (Fig. 8) and a new paragraph in the Conclusion section for the doublet rotation.

For the case of cell quadruplet rotation, the “rim-bulk graded motility” may apply transiently when there comes a “position swapping” event, in which 4 cells forming a rim-only state produces a 3 cell rim / 1 cell bulk state, which is a reminiscence of frustrated state where the cell in the core drags and slows the 3 cells in the rim. However, again, in our case there is no pre-assigned, space-dependent graded motility.

We added comments in line with this answer in the revision (lines 496-501).

Reviewer #2:

Comment: Thank you very much for answering my questions. The original submitted manuscript has no description on the mathematical model at all, and thus I could not judge the study as mentioned in my previous comments. Now the model description was provided. I found that the model is the same as one previously proposed and this study does not include any methodological novelty.

Answer #2-1:

The novelty of our computational work is NOT the Cellular Potts model itself but the phenomena and the systematic way of delineating the phenomena of pair rotation ($n=$ 2), quadruplet rotation w/ position swappings ($n=$ 4) and enhanced super-diffusivity ($n=\infty$) via the identification of a single set of parameters (s, E) in a self-consistent way. In fact, the importance of our work is the demonstration that the seemingly simple CPM is sufficient to exhibit the all the complex phenomena in a self-consistent way (as Referee #3 commented in his earlier report).

Comment: The results are quite descriptive and are not enough to convince their claim. In fact, most of the results in the manuscript (Figure 2,3,5,6) are about cell swirlings that are less directly relevant to the individual cell diffusivity.

Answer #2-2:

Again, the key to our work is delineating n=1, 2, 4, and $\infty$ cases in a systematic and self-consistent way. As we have discussed with Fig. 7, comparing n =2 and n = $\infty$ case is essential! So, it is not true that Fig. 2 ~ 6 are irrelevant for the conclusion that we draw for n = $\infty$ case.

As I commented before, quantitative description of positional swapping events in the cell clusters in experiments is interesting to some extent. However, this is not the main point for acceptance of this journal. Figure 7 shows key results for this study but it just shows parameter dependencies of produced cellular behaviors in their simplified setting. They changed the parameter value corresponding to the interfacial energy of cell-to-cell, and concluded that the cell-cell adhesion enhances the super-diffusivity of cell migrations only by means of simulations. This is caused by an enhancement of degree of cell spreading in the model, which is not solely provided by the cell-cell adhesion property in reality. The conclusion is intuitive and does not provide profound new biological insights or scientific novelty.

Overall, I do not think this study meets the criteria of this journal.

Answer #2-3:

The side-by-side comparison of the two “phase diagrams” of Fig. 7 and finding out the pair of parameters (S, E) which gives the best fit for all experimental observations in a self-consistent fashion is highly non-trivial. So, Fig. 7 is not just a parameter scan! According to the referee’s viewpoint, all phase diagrams are nothing but a parameter scan. The referee’s comment on the enhanced super-diffusivity, “The conclusion is intuitive and does not provide profound new biological insights or scientific novelty” is completely unjustifiable.

Reviewer #3: The authors' responses satisfy all my concerns, I have no further comments or criticisms for this manuscript.

Answer #3-1: Thank you for your constructive comments.
